# Assessment of Active Cytomegalovirus (CMV) and Epstein–Barr Virus (EBV) Infections and Patient Reported Fatigue in Ovarian Cancer Survivors

**DOI:** 10.1002/cnr2.70380

**Published:** 2025-11-21

**Authors:** Xuan Li, Katherine Brown, Kate Honeyfield, Devon Hunter‐Schlichting, Morgan Gruner, Mark Blackstad, Mark R. Schleiss, Deanna Teoh, Melissa A. Geller, Heather H. Nelson, Rachel I. Vogel

**Affiliations:** ^1^ Obstetrics, Gynecology and Women's Health University of Minnesota Minneapolis Minnesota USA; ^2^ Epidemiology and Community Health University of Minnesota Minneapolis Minnesota USA; ^3^ Pediatric Infectious Diseases University of Minnesota Minneapolis Minnesota USA; ^4^ Masonic Cancer Center University of Minnesota Minneapolis Minnesota USA

**Keywords:** cancer, cytomegalovirus (CMV), Epstein–Barr virus (EBV), fatigue, inflammation, survivorship

## Abstract

**Background:**

Fatigue is a common symptom reported by individuals treated for ovarian cancer. The objective of this study was to determine whether fatigue following chemotherapy for ovarian cancer is related to biomarkers of active cytomegalovirus (CMV) or Epstein–Barr virus (EBV) infection.

**Methods:**

We conducted a cross‐sectional study among individuals diagnosed with ovarian, primary peritoneal, or fallopian tube cancer in Minnesota who had completed frontline chemotherapy, irrespective of current treatment status. Participants completed a survey and provided a blood sample. The primary exposures of interest were active CMV or EBV infections, determined by measuring CMV and EBV DNA levels in plasma (DNAemia). We also assessed serology‐based markers of CMV infection and quantified levels of high‐sensitivity C‐reactive protein (hsCRP), as a biomarker of systemic inflammation. Symptoms of fatigue were self‐reported using the Fatigue Symptom Inventory. We examined associations between fatigue symptoms and CMV and EBV by infection status, with and without inflammation, using two‐sided *t*‐tests and multivariable linear regression models accounting for confounding factors.

**Results:**

Among the 160 eligible participants, 64 (40.0%) demonstrated CMV DNAemia+, 56 (35.0%) had EBV DNAemia+, and 32 (20.0%) were positive for both. There were no significant associations between CMV or EBV DNAemia and fatigue. Similarly, CMV IgG and hsCRP status, alone or combined with CMV status, were not associated with fatigue scores.

**Conclusion:**

We did not observe associations between CMV DNAemia, EBV DNAemia, or hsCRP and fatigue in this survivor population. Further investigation is needed to identify causes and indicators of cancer‐related fatigue following treatment for ovarian cancer.

## 1 Introduction

Cancer‐related fatigue and its effect on quality of life is a common distressing side effect of therapy, occurring in up to 90% of individuals receiving treatment for cancer [1]. Fatigue symptoms among those with ovarian cancer often persist after treatment completion and negatively impact mood, sleep, interpersonal relationships, and overall quality of life [2, 3]. The risk of long‐term fatigue in cancer survivors is influenced by multiple factors, including the type of cancer, treatment modalities, and individual patient characteristics. Depression, anxiety, and stress, prolonged periods of inactivity, pre‐existing health conditions such as heart disease, diabetes, and thyroid disorders, disrupted sleep patterns, and chronic inflammation during and after cancer treatment have all been associated with increased and persistent fatigue [4, 5, 6].

A study of individuals with newly diagnosed breast cancer examined whether cytomegalovirus (CMV) and Epstein–Barr virus (EBV) IgG titer levels—an indication that a person has been exposed to CMV or EBV at some point, but does not necessarily indicate current infection—were associated with fatigue at the time of diagnosis [7]. They found that fatigue at the time of diagnosis was associated with higher levels of CMV IgG, but not EBV. These results suggest that CMV reactivation may contribute to cancer‐associated fatigue. We and others have documented that active CMV infection is common among ovarian cancer patients, both at the time of diagnosis as well as during and following completion of front‐line chemotherapy for ovarian cancer [8].

In this study, we sought to build upon this previous work by examining whether there is an association between post‐treatment fatigue and active infection with CMV or EBV in a cohort of ovarian cancer survivors who have completed front‐line treatment. As a secondary aim we evaluated whether high sensitivity C‐reactive protein (hsCRP), an acute phase protein that is a downstream marker of inflammation, played a role in associations between viral infection and fatigue.

## 2 Methods

This cross‐sectional study was approved by the University of Minnesota Institutional Review Board (STUDY00013073) and registered with ClinicalTrials.gov (NCT03921658) as part of a larger study. Methods for the cross‐sectional study have previously been described in detail and are briefly summarized here [9].

Participants were recruited between October 2019 and July 2023 from cancer clinics across Minnesota. Eligible participants were 18 years of age or older, had a history of ovarian, primary peritoneal, or fallopian tube cancer, were previously treated with at least three cycles of chemotherapy for their cancer, and were able to read and write English. Individuals were eligible regardless of current disease status. Participants provided written informed consent, completed a one‐time survey, and provided a blood sample aligned with a standard‐of‐care blood draw. Participants were provided a $20 gift card for completion of study procedures.

CMV and EBV DNAemia, a biomarker of active infection, were measured using digital PCR with cell‐free DNA extracted from plasma as the starting template as described previously [10]. Samples were analyzed in duplicate, and results reported as average copy numbers/mL of plasma. Participant infection status was classified as CMV+ or EBV+ if DNAemia levels were ≥ 100 copies/mL and CMV‐ or EBV‐ if below this threshold. The hsCRP was measured using a standard FDA‐approved Roche Diagnostics assay, with levels exceeding 10 mg/dL indicating significant inflammation. CMV serology endpoints were also assessed. IgG was measured using an FDA‐approved immunoassay from Roche Diagnostics. Samples below 0.5 COI (cut off index) units were CMV negative, and those above 1.0 were CMV positive. We also measured CMV IgM using the CMV IgM kit from Gold Standard Diagnostics (Cat. No. 01‐150) according to the protocol supplied with the kit. All samples were run in duplicate and their absorbance values averaged and normalized to the reagent blank. Samples below 0.9 COI (cut off index) units were defined as IgM CMV negative, samples between 0.9 and 1.1 were equivocal (indeterminate), and values greater than or equal to 1.1 were considered IgM CMV positive.

The survey assessed numerous components of quality of life using validated surveys, symptoms and participant demographics. The primary outcome for this analysis was self‐reported fatigue, measured using the Fatigue Symptom Inventory (FSI) [11]. Severity was rated on an 11‐point Likert scale (0 = “not at all fatigued”, 10 = “as fatigued as I could be”), with higher scores indicating greater fatigue. A composite fatigue score was calculated by averaging three severity items (average fatigue, most fatigued day, least fatigued day in the past week).

To explore sleep and depression as potential confounders of the relationship between viral infection and fatigue, self‐reported sleep and depression data were collected using the PROMIS Sleep Disturbance—Short Form 6a and the Patient Health Questionnaire‐8 (PHQ‐8), respectively [12, 13]. Clinical data were abstracted from the medical record, including body mass index (BMI), International Federation of Gynecology and Obstetrics (FIGO) stage at diagnosis, histology, treatment history, diagnosis of recurrence, and current treatment status.

Demographic and exposure measures were summarized using descriptive statistics. FSI fatigue severity scores were summarized as means ± standard deviations (SD) and compared by CMV DNAemia, EBV DNAemia, CMV IgG, and hsCRP status using two‐sided *t*‐tests. Multivariable linear regression models examined associations between each exposure and fatigue, adjusting for age, BMI, depression symptoms, sleep, FIGO stage (I/II, III/IV), time since diagnosis, and treatment status (no treatment/surveillance, maintenance therapy, treatment for progression/recurrence). We further explored potential subgroups of interest, stratifying by hsCRP result, recurrence status and recency of receipt of chemotherapy prior to participating in the study (< 30 days, 1–12 months, 1–2 years, 2+ years). Analyses were conducted using SAS version 9.4, with *p*‐values ≤ 0.05 considered statistically significant.

## 3 Results

A total of 326 potentially eligible participants were approached to participate in this study, 200 (61.3%) consented, and 160 (49.1%) completed the survey within 14 days of the blood draw. Participants were a median of 64 (range: 25–92) years old and were primarily non‐Hispanic White. Approximately half (52.2%) of participants were college graduates (Table 1). Participants were a median of 2.2 years from diagnosis, the majority had advanced stage disease and 43.1% experienced at least one recurrence prior to participating.

**TABLE 1:** Participant characteristics (*N* = 160).

Characteristic	Median (Min, Max)
Age (years)	64 (25–92)
BMI (kg/m^2^)	29.0 (17.5–49.3)
Time since diagnosis (years)	2.2 (0.3–22.3)
Number of chemotherapy cycles before survey	8 (3–57)

Characteristic	*N* (%)
Race	
Asian	3 (1.9)
Black or African American	4 (2.5)
White	150 (94.9)
More than one	1 (0.6)
*Not reported*	*2*
Ethnicity	
Hispanic or Latino	6 (3.8)
Not Hispanic/Latino	153 (96.2)
*Not reported*	*1*
Education	
No college	76 (47.8)
College graduate	83 (52.2)
*Not reported*	*1*
Annual household income	
< $50,000	39 (24.8)
$50 000–$99 999	46 (29.3)
$100 000+	47 (29.9)
Prefer not to answer	25 (15.9)
*Not reported*	*3*
Marital status	
Never married	27 (17.1)
Married/partnered	101 (63.9)
Widowed	13 (8.2)
Divorced	17 (10.8)
*Not reported*	*2*
Cancer stage	
Early stage (I/II)	39 (24.8)
Advanced stage (III/IV)	118 (75.2)
*Unknown*	*3*
Recurrence prior to study	
No	91 (56.9)
Yes	69 (43.1)
Treatment status at time of survey	
No treatment/surveillance	56 (35.0)
Maintenance therapy	50 (31.3)
Treatment for progression/recurrence	54 (33.8)

*Note:* Italics indicate the data that were missing and did not contribute to the calculation of percentages.

A total of 64 (40.0%) had demonstrable CMV DNAemia; 56 (35.0%) had EBV DNAemia; and 32 (20.0%) tested positive for both viruses. Over half (93, 58.1%) were seropositive for CMV IgG antibodies. A total of 24 (15.0%) had a hsCRP of 10 or greater, indicating clinically significant inflammation. Six patients (3.8%) had CMV DNAemia and a hsCRP level of 10 mg/L or greater. Only one participant tested CMV IgM positive; this participant was negative for CMV IgG and DNAemia.

**FIGURE 1:**
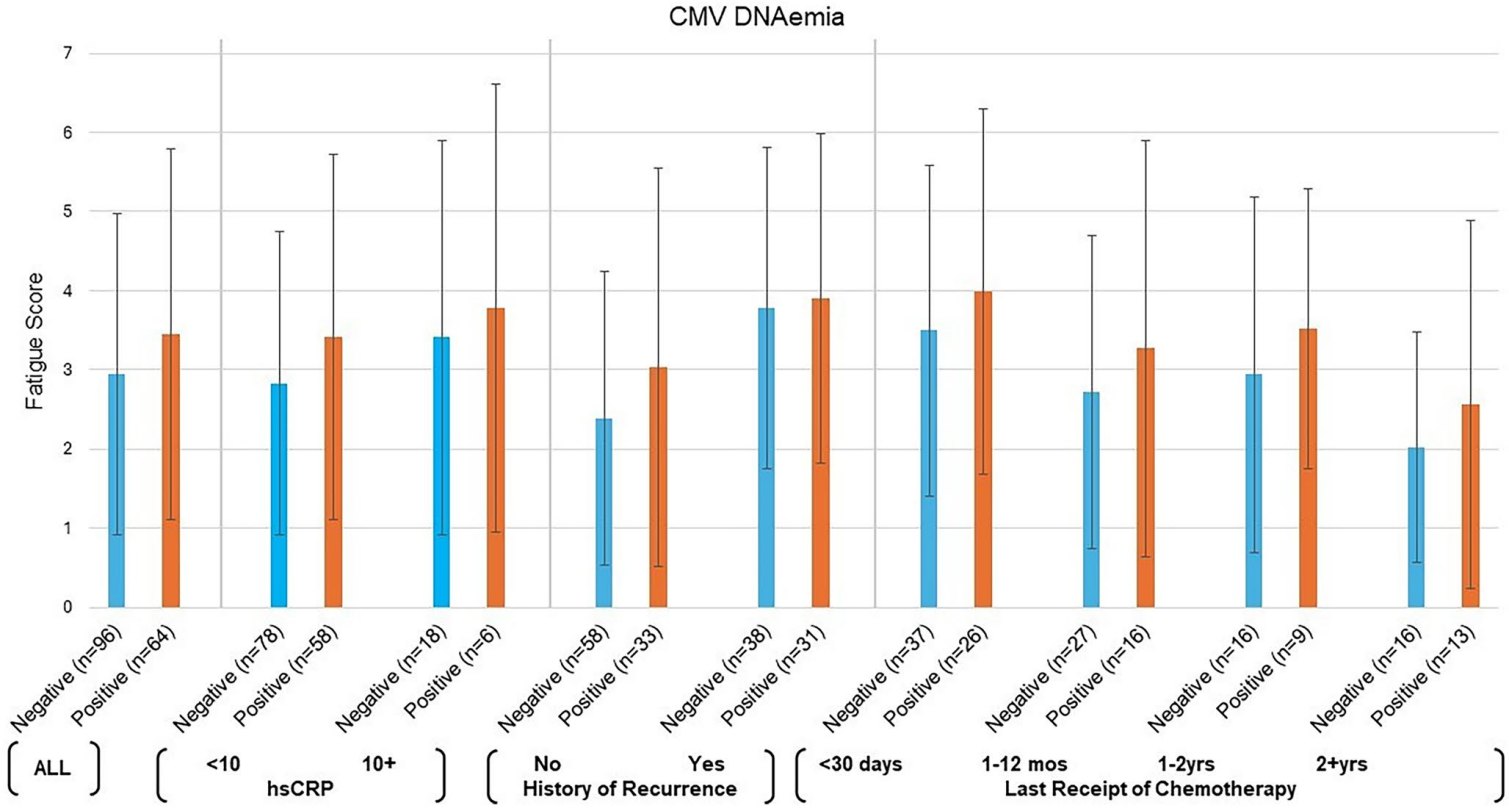
Fatigue scores by CMV DNAemia (positive, negative) for all and among subgroups based on hsCRP, recurrence status and time since last receipt of chemotherapy at the time of study participation.

The mean FSI score in the cohort was 3.1 ± 2.2 on a possible scale of 0–10. We did not observe a statistically significant association between CMV DNAemia and fatigue scores (CMV DNAemia negative: 2.9 ± 2.0 vs. CMV DNAemia positive: 3.5 ± 2.3; *p*‐value from multivariate model = 0.65; Table 2). The results were consistent (higher fatigue scores in CMV DNAemia positive group with no statistically significant associations observed in multivariate models) when exploring associations across groups of interest based on inflammation status, time since receipt of chemotherapy, or recurrence status (Figure 1). Further, CMV IgG, EBV DNAemia, or hsCRP status were not associated with fatigue scores (Table 2).

**TABLE 2:** Fatigue Symptom Inventory score by CMV, EBV and hsCRP status (*N* = 160).

	Fatigue score		Bivariate		Multivariate	
	*N*	Mean (SD)	Difference Means (95% CI)	*p*	Difference Means (95% CI)	*p* ^a^
CMV DNAemia						
Negative (< 100 copies/mL)	96	2.94 (2.03)	−0.52 (−1.20, 0.17)	0.14	−0.11 (−0.57, 0.36)	0.65
Positive (100+ copies/mL)	64	3.45 (2.34)				
CMV IgG						
Non‐reactive	67	3.27 (2.17)	0.21 (−0.47, 0.90)	0.54	0.22 (−0.25, 0.69)	0.36
Reactive	93	3.05 (2.18)				
EBV DNAemia						
Negative (< 100 copies/mL)	104	3.22 (2.17)	0.21 (−0.50, 0.92)	0.56	0.15 (−0.35, 0.64)	0.56
Positive (100+ copies/mL)	56	3.01 (2.19)				
hsCRP						
< 10	136	3.08 (2.10)	−0.42 (−1.37, 0.53)	0.38	0.25 (−0.38, 0.89)	0.43
10+	24	3.50 (2.52)				

*Note:* A higher FSI score indicates worse symptoms of fatigue.

^a^ Adjusted for age, body mass index, depression, sleep score, disease stage (I/II vs. III/IV), time since diagnosis (years), treatment status (no treatment/surveillance vs. maintenance therapy vs. treatment for progression/recurrence).

## 4 Discussion

Cancer‐related fatigue is a distressing and persistent concern for patients during and following cancer treatment, highlighting the need to understand underlying mechanisms. In prior work we have observed that when patients with ovarian cancer have CMV DNAemia they are more likely to have symptoms indicative of chemotherapy related cognitive decline and possibly peripheral neuropathy [9]. Here we have evaluated whether CMV DNAemia is associated with self‐reported fatigue. In this cross‐sectional study among ovarian cancer survivors beyond initial chemotherapy, we did not observe associations between CMV, EBV, or hsCRP status and self‐reported fatigue.

We compared our findings to a previous study of individuals diagnosed with breast cancer, which found that among newly diagnosed patients (prior to starting chemotherapy) those reporting fatigue had higher CMV antibody titers at the time of diagnosis [7]. The authors' interpretation of these findings was that higher IgG titer was suggestive of active infection, which in turn may have contributed to cancer‐related fatigue. Our study evaluated CMV status and fatigue post‐treatment, whereas the Fagundes study explored relationships between CMV and fatigue pre‐treatment [7]. Of note, our prior pilot work on CMV infection and cancer related cognitive impairment indicated CMV status at the time of diagnosis was a better predictor of impairment than CMV measured during or post treatment [8]. It is also possible that the heterogeneity in our study population with respect to time since diagnosis and treatment status may have impaired our ability to observe an association. These differences may reflect differences in the contribution of virus to fatigue at the time of cancer diagnosis versus following cancer treatment, or differences between the study populations. It is also possible that the etiology of cancer fatigue is etiologically distinct in breast and ovarian cancer.

Cancer‐related fatigue is a complex, multifactorial syndrome that is likely caused by a cascade of biological dysregulations. Though not well understood, current research points to three primary, interconnected biological pathways: chronic systemic inflammation, dysfunction of the hypothalamic–pituitary–adrenal (HPA) axis, and impaired mitochondrial function [5, 14]. These mechanisms are not isolated but rather likely interact. Sleight et al. recently proposed leveraging the 3P (predisposing, precipitating, and perpetuating) factors model as a framework to understand the etiology of cancer‐related fatigue [15]. Under this model, it is possible that CMV viral infection, for example, might be a precipitating factor and in this survivor population it is not a perpetuating factor that prolongs fatigue symptoms. CMV infection status has been reported in non‐oncology patients to be negatively associated with diminished physical function and quality of life, including fatigue [16]. Further research is needed to more comprehensively understand the mechanisms and predisposing, precipitating and perpetuating factors of cancer‐related fatigue.

The strengths of this study include a relatively large sample of ovarian cancer survivors and our ability to account for multiple potential confounding factors. However, this study has notable limitations. First, it was a cross‐sectional study and therefore both the exposures and outcomes were assessed at the same time point, resulting in a lack of information on infection timing and precluding conclusions regarding causation. Second, there is potential for residual confounding from other unmeasured variables such as comorbidities, physical activity, or other medication use. Third, while inflammation was not the focus of this analysis, hsCRP was the only marker of inflammation assessed in this study; other more specific markers such as IL‐6 or TNF‐α may provide more insight. Fourth, CMV has been detected at low copy numbers in some ovarian tissue samples [17, 18, 19, 20]. We have not evaluated whether the infection we are detecting is from the tumor or a generalized systemic infection. Additionally, the sample was predominantly non‐Hispanic White, limiting the generalizability of the results; future research needs to include diverse participants.

In conclusion, we did not observe associations between CMV infection, EBV infection, or hsCRP and fatigue in this population of patients with ovarian, primary peritoneal, or fallopian tube cancer post‐frontline therapy. Further research is needed to identify risk factors and mechanisms of persistent fatigue among ovarian cancer survivors.

## Author Contributions

**Xuan Li:** data curation, writing – original draft, writing – review and editing. **Katherine Brown:** data curation, project administration, writing – review and editing. **Kate Honeyfield:** data curation, writing – review and editing. **Devon Hunter‐Schlichting:** data curation, formal analysis, writing – review and editing. **Morgan Gruner:** data curation, writing – review and editing. **Mark Blackstad:** data curation, formal analysis, writing – review and editing. **Mark R. Schleiss:** formal analysis, writing – review and editing. **Deanna Teoh:** investigation, writing – review and editing. **Melissa A. Geller:** conceptualization, data curation, investigation, supervision, writing – review and editing. **Heather H. Nelson:** conceptualization, data curation, formal analysis, investigation, methodology, supervision, writing – original draft, writing – review and editing. **Rachel I. Vogel:** conceptualization, data curation, formal analysis, funding acquisition, methodology, project administration, supervision, writing – original draft, writing – review and editing.

## Conflicts of Interest

The authors declare no conflicts of interest.

## Data Availability

The data that support the findings of this study are available on request from the corresponding author and review by the University of Minnesota Institutional Review Board. The data are not publicly available due to privacy or ethical restrictions.
